# RUNX3 Promotes the Tumorigenic Phenotype in KGN, a Human Granulosa Cell Tumor-Derived Cell Line

**DOI:** 10.3390/ijms20143471

**Published:** 2019-07-15

**Authors:** Huachen Chen, Powel Crosley, Abul K. Azad, Nidhi Gupta, Nisha Gokul, Zhihua Xu, Michael Weinfeld, Lynne-Marie Postovit, Stephanie A. Pangas, Mary M. Hitt, YangXin Fu

**Affiliations:** 1 Department of Oncology, Faculty of Medicine and Dentistry, University of Alberta, Edmonton, AB T6G 2E1, Canada; 2 Department of Pathology & Immunology and Department of Molecular & Cellular Biology, Baylor College of Medicine, Houston, TX 77030, USA; 3 Graduate Program in Molecular & Cellular Biology, Baylor College of Medicine, Houston, TX 77030, USA; 4 Department of Obstetrics and Gynecology, Faculty of Medicine and Dentistry, University of Alberta, Edmonton, AB T6G 2E1, Canada

**Keywords:** granulosa cell tumor of the ovary, KGN, COV434, RUNX3, cyclin D2, p27^Kip1^

## Abstract

Granulosa cell tumors of the ovary (GCT) are the predominant type of ovarian sex cord/stromal tumor. Although prognosis is generally favorable, the outcome for advanced and recurrent GCT is poor. A better understanding of the molecular pathogenesis of GCT is critical to developing effective therapeutic strategies. Here we have examined the potential role of the runt-related transcription factor RUNX3. There are only two GCT cell lines available. While RUNX3 is silenced in the GCT cell line KGN cells, it is highly expressed in another GCT cell line, COV434 cells. Re-expression of RUNX3 promotes proliferation, anchorage-independent growth, and motility in KGN cells in vitro and tumor formation in mice in vivo. Furthermore, expression of a dominant negative form of RUNX3 decreases proliferation of COV434 cells. To address a potential mechanism of action, we examined expression of cyclin D2 and the CDK inhibitor p27^Kip1^, two cell cycle regulators known to be critical determinants of GCT cell proliferation. We found that RUNX3 upregulates the expression of cyclin D2 at the mRNA and protein level, and decreases the level of the p27^Kip1^ protein, but not p27^Kip1^ mRNA. In conclusion, we demonstrate that RUNX proteins are expressed in GCT cell lines and human GCT specimens, albeit at variable levels, and RUNX3 may play an oncogenic role in a subset of GCTs.

## 1. Introduction

Granulosa cell tumors of the ovary (GCT) are the predominant type of ovarian sex cord/stromal tumors that account for 3–5% of all malignant ovarian tumors [1]. Although prognosis is generally favorable, the outcome for advanced and recurrent GCT is poor [1,2,3]. The rate of GCT recurrence has been reported to be 10–28% and approximately 80% of patients who relapse will succumb to disease [3,4,5]. Surgery is the predominant form of treatment for patients with early stage disease, and chemotherapy is used to treat recurrent or metastatic disease [1,3,4]. However, there is no standard treatment for relapsed GCT patients. A better understanding of the molecular pathogenesis of GCT is critical to developing effective therapeutic strategies. GCT is believed to arise from proliferating granulosa cells of the periovulatory follicle. It is classified into two distinct forms: Adult GCT (AGCT) and juvenile GCT (JGCT) with AGCT accounting for 95% of GCT cases [4]. AGCT and JGCT present distinct genetic alterations, pathological features, and clinical behaviors [4,6]. A somatic missense mutation, 402C>G (C134W), in the *FOXL2* gene was identified in 97% of AGCT, but not in JGCT [7]. Mutant FOXL2 retains some functions of wild-type FOXL2, but also shows altered functions, suggesting a role for mutated FOXL2 in the pathogenesis of AGCT [8,9,10].

Runt-related transcription factors (RUNX1–3) play an important role in normal tissue development and in cancer [11,12,13]. RUNX proteins bind to a specific DNA sequence and form a heterodimer with CBFβ/PEBP2β (core-binding factor-β subunit/polyomavirus enhancer-binding protein 2 β subunit) to regulate the expression of their target genes [14]. Studies in recent years using in vitro and in vivo models in mice and rats have revealed a critical role for RUNX proteins and CBFβ in granulosa cells. *Runx1* and *Runx2* are induced by luteinizing hormones (LH) in periovulatory granulosa cells and concurrently regulate gene expression in luteinizing granulosa cells during ovarian folliculogenesis [15,16,17,18]. *Runx3* is also expressed in granulosa cells and regulates folliculogenesis and steroidogenesis in granulosa cells of mice; Runx3 knockout mice are anovulatory [19,20]. Granulosa cell-specific knockout of *Cbfb*, which encodes the heterodimeric partner of RUNX proteins, CBFβ, alters the expression of the genes in ovulatory follicles and results in loss of fertility in female mice [21,22]. These studies indicate that RUNX proteins, as well as CBFβ, concurrently regulate the expression of the genes that are involved in the ovulation of follicles and differentiation of luteinizing granulosa cells, and thus regulate ovulation and luteal development and function.

RUNX proteins are either tumor suppressors or oncogenes in human cancers [23]. Accumulating evidence indicates that RUNX proteins and CBFβ play an oncogenic role in epithelial ovarian cancer (EOC); they promote growth, migration, and invasion, as well as carboplatin resistance, in EOC [24,25,26,27,28,29]. Despite the well-documented functions of RUNX proteins and CBFβ in the normal physiology of granulosa cells, it is unknown whether they play a role in pathogenesis of GCT. One previous study showed that the promoter of RUNX3 is methylated, resulting in the silencing of its expression in 56% of human GCT [30]. In the present study, we examined the expression of RUNX proteins and CBFβ in human GCT cell lines and determined that RUNX3 promotes the tumorigenic phenotypes in GCT cells.

## 2. Results

### 2.1. Expression of RUNX Proteins in GCT and Immortalized Granulosa Cell Lines

**Figure 1:**
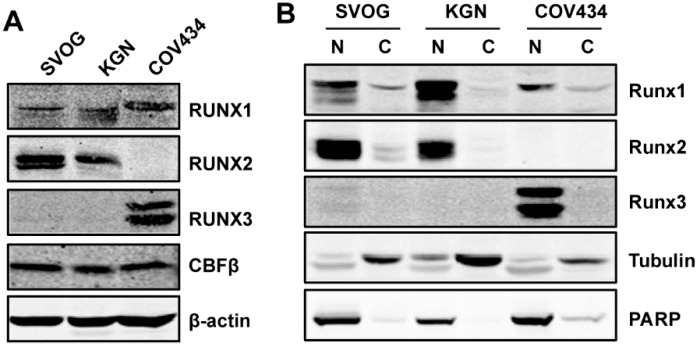
Expression of runt-related transcription factor (RUNX) proteins in immortalized granulosa and granulosa cell tumors of the ovary (GCT) cell line. (**A**) The expression of RUNX proteins and core-binding factor-β subunit (CBFβ) in SVOG (immortalized human granulosa), KGN (adult GCT), and COV434 (juvenile GCT) cells was examined by immunoblotting. β-actin was used as the loading control. (**B**) Nuclear localization of RUNX proteins was confirmed using subcellular fractionation and immunoblotting. Tubulin and PARP were used as loading and fractionation controls for the cytosolic and nuclear fractions, respectively. N: Nuclear fraction; C: Cytosolic fraction.

Currently, there are only two GCT cell lines available: KGN (an AGCT cell line) and COV434 (a JCGT cell line). To determine whether RUNX proteins are involved in pathogenesis of GCT, we first examined their expression in the two GCT cell lines and an immortalized human granulosa cell line (SVOG). Immunoblotting showed that RUNX1 and CBFβ were expressed in all cell lines, whereas RUNX2 was expressed in KGN and SVOG, but not in COV434 (Figure 1A). RUNX3 was expressed at a high level in COV434 cells, but was not detectable in SVOG and KGN cells (Figure 1A). The two bands of RUNX3 in COV434 cells likely correspond to the two major isoforms of RUNX3 (p44 and p46), as observed in other types of cells [24,29,31]. All three RUNX proteins were predominantly localized to the nucleus of these cells (Figure 1B), which is consistent with the function of RUNX proteins as transcription factors.

### 2.2. RUNX3 Promotes Cell Growth and Migration in KGN Cells

**Figure 2:**
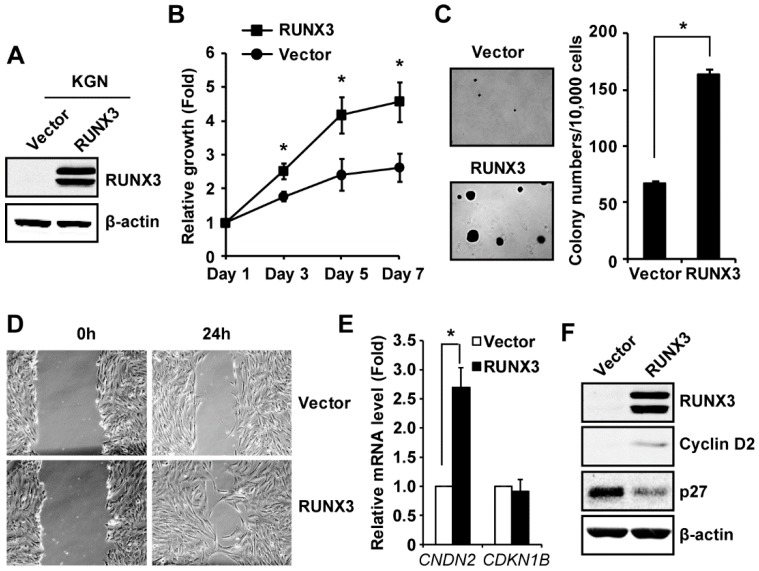
RUNX3 promotes the tumorigenic phenotypes of KGN cell in vitro. (**A**) Ectopic expression of RUNX3 in KGN cells was examined by immunoblotting. β-actin was used as the loading control. (**B**) Cell growth was determined by the neutral red uptake assay and expressed as the fold change relative to day 1. (**C**) Anchorage-independent growth was examined by the soft agar assay and the number of colonies formed by KGN/Vector and KGN/RUNX3 cells were counted. (**D**) Cell motility was determined by the scratch assay. Images were captured under the phase contrast microscope at 100× magnification. (**E**) The mRNA level of *CCND2* (cyclin D) and *CDK1B* (p27) was measured by quantitative reverse transcription-PCR (qRT-PCR) and expressed as the fold change relative to the vector-only control cells. (**F**) Cyclin D2 and p27^Kip1^ protein levels were examined by immunoblotting. β-actin was used as the loading control. Data in (**B**,**C**,**E**) are shown as mean ± SE of three independent experiments. * Significantly different (*p* < 0.05). Results in (**D**) and (**F**) are representative of three independent experiments.

Our finding that RUNX3 is highly expressed in COV434 cells prompted us to examine whether aberrant RUNX3 expression contributes to the tumorigenesis of GCT. One previous study showed that the promoter of the *RUNX3* gene is methylated in 15 out of 25 human GCT tissues and in KGN cells [30], causing RUNX3 silencing. However, that study did not investigate the biological function of RUNX3 in GCT. To address this question, we stably transduced KGN cells with an empty vector or a vector expressing RUNX3-FLAG (RUNX3 protein was FLAG tagged) to generate KGN/Vector and KGN/RUNX3 cells as we have done previously [29]. Ectopic expression of RUNX3 in KGN cells was confirmed by immunoblotting, using an anti-RUNX3 antibody (Figure 2A). Expression of two RUNX3 bands by this vector is consistent with studies published by others and us [24,29,31]. Functional assays showed that RUNX3 increased cell growth (Figure 2B), colony formation in soft agar (measurement of cellular transformation) (Figure 2C), and motility of KGN cells (Figure 2D). Quantification of the scratch assay results of three independent experiments showed RUNX3 increased the motility of KGN cells by 59% (*n* = 3, *p* < 0.05). Taken together, our results indicate that expression of RUNX3 promotes the in vitro tumorigenic phenotypes in KGN cells.

### 2.3. RUNX3 Regulates the Expression of Cyclin D2 and CDK Inhibitor p27^Kip1^ in KGN Cells

Two cell cycle regulators, Cyclin D2 and CDK inhibitor p27^Kip1^, are involved in the proliferation and survival of GCT cells [32,33,34] and the balance between cyclin D2 and p27^Kip1^ has been shown to determine the proliferation and differentiation of granulosa cells [35]. Our immunoblotting showed that RUNX3 upregulated the expression of cyclin D2 at both the mRNA (by 2.7 times, Figure 2E) and protein (Figure 2F) levels in KGN cells. Interestingly, RUNX3 decreased p27^Kip1^ protein by 58% (*n* = 3, *p* < 0.05) (Figure 2F) without changing its mRNA level in KGN cells (Figure 2E). Our results suggest that RUNX3 increases proliferation by regulating the expression of cell cycle regulators in KGN cells.

### 2.4. RUNX3 Enables KGN Cells to Form Subcutaneous Tumors

**Figure 3:**
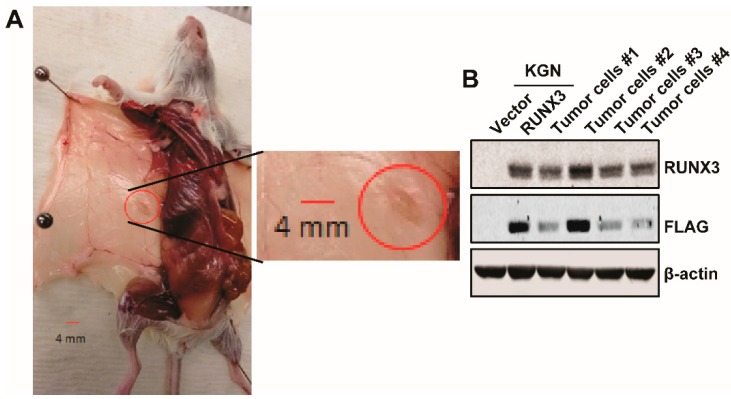
RUNX3 increases tumor formation in KGN cells. KGN/Vector and KGN/RUNX3 cells (2 × 10^7^ cells) were injected subcutaneously into the left and right flank, respectively, of female NSG (NOD-*scid* IL2R-gamma^null^) mice twice over an interval of two weeks (*n* = 6). (**A**) Image of one KGN/RUNX3 tumor is shown. The left panel shows the location of the tumor. The right panel shows the image of the same tumor at a higher magnification. The scale bar is 4 mm. (**B**) Four KGN/RUNX3 tumors were harvested, dissociated into single cells, and passaged in culture. RUNX3 expression in the tumor-derived cells was examined by immunoblotting using RUNX3 and FLAG antibodies. β-actin was used as the loading control.

KGN cells have been shown to have a limited capacity to form xenografts in mice [36,37,38]. To determine whether RUNX3 promotes KGN tumor formation in a mouse xenograft model, we subcutaneously injected KGN/Vector and KGN/RUNX3 cells into contralateral flanks of female NSG (NOD-*scid* IL2R-gamma^null^) mice and monitored tumor formation twice a week by palpation for up to 185 days. Interestingly, four out of six injections of KGN/RUNX3 cells, but none of the six KGN/Vector injections, formed small tumors (Figure 3A). We then dissociated tumors into single cells and expanded them in culture. Cells from all four tumors proliferated well in culture and displayed the same morphology as unmodified KGN/RUNX3 cells (Supplementary Materials, Figure S1). We confirmed that these tumor-derived cells were indeed KGN/RUNX3 cells by detecting expression of RUNX3-FLAG, as determined by immunoblotting, using a specific RUNX3 antibody or an anti-FLAG antibody (Figure 3B), as well as by quantitative reverse transcription-PCR (qRT-PCR) using primers specific to human RUNX3 (data not shown). Our results suggest that expression of RUNX3 increases the capacity of KGN cells to form tumors in mice.

### 2.5. Overexpression of dnRUNX3 Decreases Growth of COV434 Cells

**Figure 4:**
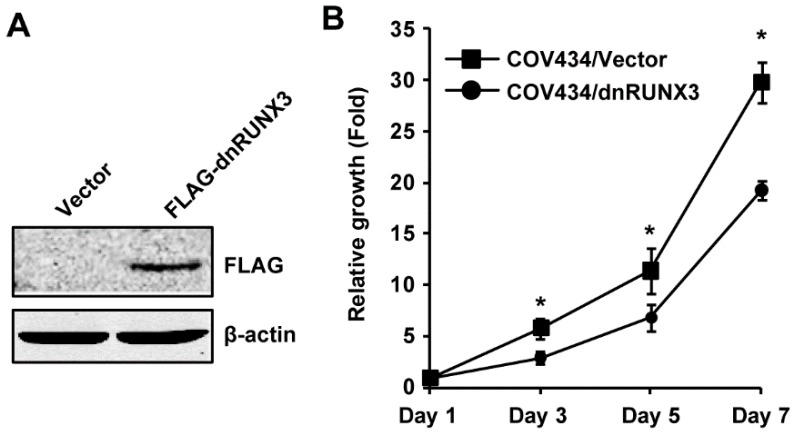
Inhibition of RUNX3 by a dominant–negative form of RUNX3 decreases growth of COV434 cells. (**A**) Expression of RUNX3 (1–187), a truncated RUNX3 that functions as a dominant–negative RUNX3 (dnRUNX3), was confirmed by immunoblotting using a FLAG antibody. β-actin was used as the loading control. (**B**) DnRUNX3 decreased cell growth of COV434 cells as determined by the neutral red uptake assay. The relative cell growth in each cell type was normalized to their respective day 1 controls. Data are shown as mean ± SE of three independent experiments. * Significantly different (*p* < 0.05).

Because COV434 cells express a high level of RUNX3, we wanted to determine whether the elevated expression of RUNX3 affects the behavior of COV434 cells. We stably transfected COV434 with an empty vector pcDNA3.1 or pCDNA-FLAG-RUNX3 (1–187) (a dominant negative form of RUNX3, dnRUNX3) to generate COV434/Vector and COV434/dnRUNX3 cells. Expression of dnRUNX3 was confirmed by immunoblotting, using a FLAG antibody (Figure 4A). The neutral red uptake assay showed that the inhibition of RUNX3 by dnRUNX3 decreased proliferation of COV434 cells (Figure 4B), which is in line with increased cell growth caused by RUNX3 re-expression in KGN cells.

### 2.6. Expression of RUNX Proteins in Human GCT Tissues

**Figure 5:**
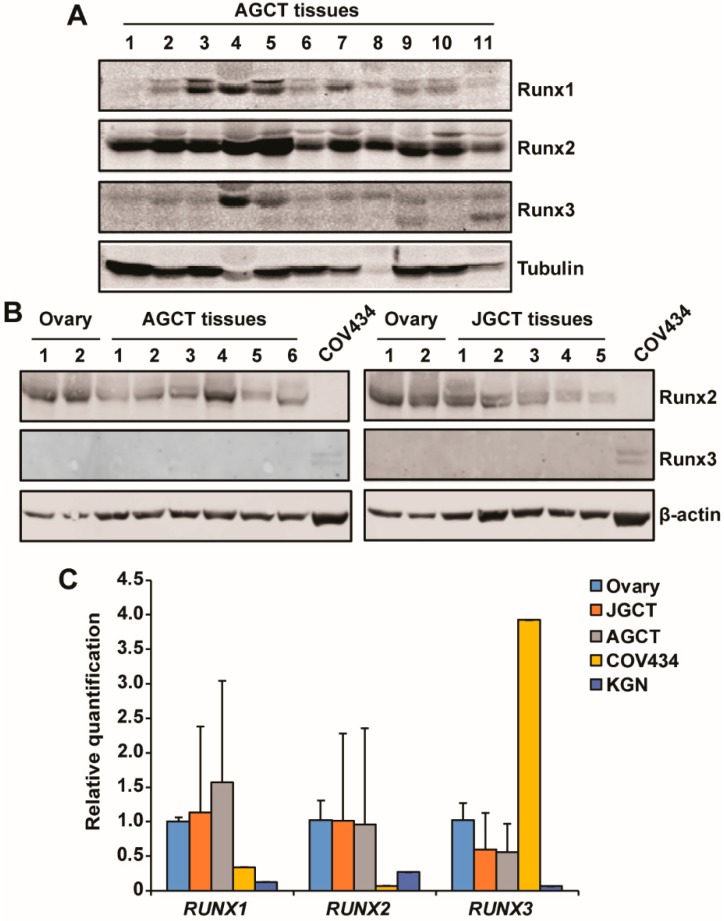
Expression of RUNX proteins in human GCT tissues. (**A**) The expression of RUNX proteins in human adult GCT tissues was examined by immunoblotting. Tubulin was used as the loading control. (**B**) The expression of RUNX2 and RUNX3 in human adult and juvenile GCT tissues, as well as the normal ovary, was examined by immunoblotting. β-actin was used as the loading control. (**C**) The mRNA level of RUNX1, RUNX2, and RUNX3 in human adult and juvenile GCT tissues, as well as the normal ovary, was examined by qRT-PCR and expressed as the relative quantification against that in the normal ovary. Data for the normal ovary and GCT tissues are shown as mean ± SD (*n* = 3 for ovary; *n* = 5 for JGCT; and *n* = 6 for AGCT).

Next, we examined the expression of RUNX proteins in samples of adult and juvenile human GCT tissues. Immunoblotting showed that RUNX2 was expressed at a high level and RUNX1 at a variable level in 11 AGCT samples from the Alberta Cancer Research Biobank. In contrast, we detected RUNX3 at low levels in 11 AGCT tissues obtained from the Alberta Cancer Research Biobank, but in none of the six AGCT from Baylor College of Medicine Tissue Repository (Figure 5A,B). Similarly, RUNX2 expression, but not RUNX3, was detected in all three normal ovarian tissues and all five JGCT samples obtained from Baylor College of Medicine (Figure 5B). qRT-PCR detected a variable mRNA level of RUNX1–3 in the samples from the Baylor College of Medicine (Figure 5C). Consistent with the immunoblotting results shown in Figure 1A, COV434 cells expressed the lowest level of RUNX2 and the highest level of RUNX3 among the samples (Figure 5C). Taken together, our results suggest that expression of RUNX3 may not be a common event in human GCT.

## 3. Discussion

Multiple signaling pathways that are involved in the biology of normal granulosa cells are implicated in the pathogenesis of GCT [4]. RUNX1–3 and CBFβ are critical factors in regulating the biology of granulosa cells [15,16,17,18,19,20,21,22]; however, their role in tumorigenesis of GCT is unknown. In this study, we found that RUNX1, RUNX2, and CBFβ are expressed in SVOG and KGN cells, but RUNX2 expression is lost and RUNX3 is highly expressed in the JGCT cell line, COV434 cells. We therefore wanted to determine whether RUNX3 is implicated in the pathogenesis of GCT, in particular, JGCT. Because KGN is the only other GCT cell line available nowadays, we wanted to determine the impact of RUNX3 expression on the behavior of this cell line. A previous study showed that the promoter of the *RUNX3* gene was methylated in 14 out of 25 (56%) GCT patient tissues, as well as in KGN cells. *RUNX3* mRNA became detectable in KGN cells after 5-aza-2′-deoxycytidine (a demethylating agent) treatment [30] as further evidence that RUNX3 is silenced by promoter methylation. In addition to promoter methylation, RUNX3 expression and function are also regulated via posttranslational modifications, including phosphorylation, acetylation, and ubiquitination [39,40,41]. RUNX3 can be a tumor suppressor or an oncogene, depending on the cancer type [23]. In this study, we demonstrate that re-expression of RUNX3 in KGN cells promotes cell growth, colony formation, and migration in vitro, as well as tumor formation in mice, which likely involves regulating the expression of cyclin D2 and p27^Kip1^, two key cell cycle regulators in GCT [32,33,34]. Our results thus suggest that RUNX3, when expressed, could play an oncogenic role in GCT. Mechanistically, RUNX3 upregulates cyclin D2 at the mRNA level, whereas RUNX3 over-expression results in down-regulation of p27^Kip1^, likely via post-translational regulation, because RUNX3 decreases p27^kip1^ protein level without changing its mRNA level in KGN cells. Indeed, it has been well documented that although p27^Kip1^ expression is regulated by both transcriptional and post-translational mechanisms, post-translational modification that alters E3 ubiquitin ligase-mediated protein degradation is the predominant mechanism to control p27^kip1^ protein level [42]. Additional experiments are warranted to define the mechanisms by which RUNX3 regulates cyclin D2 and p27^Kip1^ expression in GCT in detail.

In general, the majority of GCT cases are diagnosed at the early stages when tumors are still localized and indolent [1,2,3]. With surgery as the standard treatment, the 10-year survival rate for stage I GCT can be up to 96% [1,2,3]. However, the outcome for advanced and recurrent GCT is poor [1,2,3]. It has been reported that the rate of GCT recurrence is 10–28% and approximately 80% of patients who relapse will succumb to disease [3,4,5]. Beyond surgery, there is no standard treatment for recurrent GCT patients and current therapeutic regimes are ineffective in treating recurrent GCT. Development of novel therapies is hindered by the lack of a preclinical in vivo model for this disease [43]. Thus far, only a few studies reported mouse xenograft models using KGN cells, and in these studies, KGN cells formed small tumors with delayed growth [36,37,38]. It is our future interest to develop mouse xenograft models by repeated in vivo passaging of these KGN/RUNX3 tumors in mice, in order to establish a KGN xenograft model with a faster tumor growth that would be suitable for therapeutic drug testing [43].

Our results show that expression of a dominant–negative form of RUNX3 decreased cell growth in COV434 cells. However, a recent study suggested that COV434 cells may be misidentified and are possibly a small cell carcinoma of the ovary, hypercalcemic type (SCCOHT) cell line [44]. Additionally, the RUNX2 and RUNX3 expression patterns in COV434 do not seem to be representative of the primary JGCT samples examined here, although these were few in number. Because of the current uncertainty in the origin of COV434 cells and until there is further analysis of that cell line, we are limited in our ability to interpret these data with respect to JGCT development.

Another observation was the reciprocal relationship between RUNX2 and RUNX3 expression in COV434 and KGN cells. Whether there is a mutual negative regulation between these two transcription factors would be interesting to examine. Furthermore, our results show that RUNX2 was expressed in all the AGCT and JGCT samples we examined, suggesting that expression of RUNX2 could be an important event in GCT development, and should be further investigated.

One critical question is the importance of RUNX3 in human AGCT, given its variable expression in our study and others [30]. Our immunoblotting and qRT-PCR results show that RUNX3 was only weakly produced in many of the human AGCT samples and not at all in JGCT tissues that we tested. Although β-actin loading was not very even in Figure 5B, as we were not quantitatively comparing the expression of RUNX2/3 between the samples, the β-actin blot was adequate to serve as a loading control to indicate that RUNX2, but not RUNX3, was detected in these human GCT tissues. GCT is a rare tumor and it is very hard to collect human GCT tissues for research, especially the JGCT tissues. Re-running the immunoblotting using the rare and limited quantity samples to get more even β-actin will not change the outcome of the study. Our data suggest that ectopic RUNX3 expression promotes tumorigenicity, but whether it plays an important role in the development of GCT awaits a better understanding of the function of RUNX3 in normal human ovaries. It is possible that RUNX3 may have a folliculogenesis stage-specific or limited expression pattern in human granulosa cells that is not reflected in KGN or the transformed SVOG cell line. Our findings that RUNX3 promotes tumorigenic phenotypes in KGN cells and that RUNX3 is expressed in some AGCT tissues, which are consistent with the report by Dhillon et al. [30], suggest that RUNX3 might play an oncogenic role in a subset of GCT. However, due to the rarity of GCT and the limited availability of human GCT tissues for research, only small sample sizes were analyzed in this study. Additional sample collection via collaboration of multiple institutions, along with broader functional studies, is required to fully uncover any potential role of RUNX3 in the etiology of GCT.

In summary, we demonstrate that RUNX proteins are expressed in GCT cell lines and human GCT specimens, albeit at varying levels, and RUNX3 may play an oncogenic role in a subset of GCT. A recent study showed that targeting RUNX/CBFβ interaction using small molecule inhibitors decreased cell proliferation, motility, and anchorage-independent growth in EOC, suggesting that inhibition of RUNX/CBFβ function could be a potential therapeutic strategy to treat EOC [45]. However, because EOC and GCT are different in cells of origin, pathophysiology, and response to chemotherapy, a better understanding of the role for RUNX/CBFβ in tumorigenesis of GCT is warranted to determine whether targeting RUNX/CBFβ could be a potential therapeutic strategy to treat GCT.

## 4. Materials and Methods

### 4.1. Reagents

Primary antibodies against RUNX1 (#4334), RUNX2 (#8486), p27^Kip1^ (#3698), and PARP (#9532) were purchased from Cell Signaling Technology (Danvers, MA, USA); antibodies against RUNX3 (ab40278), Cyclin D2 (ab207604), and tubulin (ab59680) were purchased from Abcam (Toronto, ON, Canada); antibodies against FLAG (F1804) and β-actin antibody (A5441) were purchased from Sigma–Aldrich (Oakville, ON, Canada); and the antibody against PEBP2β (CBFβ) was purchased from Santa Cruz Biotechnology (Dallas, TX, USA). Secondary antibody IRDye 800CW conjugates of donkey anti-rabbit-IgG were purchased from LI-COR Biosciences (Lincoln, NE, USA). Neutral red dye was purchased from Sigma–Aldrich (Oakville, ON, Canada).

### 4.2. Cell Culture and Gene Transfer

KGN, an AGCT cell line that was developed from a stage III primary adult GCT tumor [46], was purchased from the RIKEN Cell Bank and cultured in DMEM/F12 medium. COV434, a JGCT cell line [47], was purchased from Sigma (07071909, Oakville, ON, Canada) and cultured in DMEM medium. The immortalized granulosa cell line SVOG was obtained from the Canadian Ovarian Cell Bank at the BC Cancer Research Centre and cultured in MCDB105/M199 (1:1) in the presence of 0.4 μg/mL hydrocortisone. All medium was supplemented with 10% FBS, 100 U/mL penicillin, and 100 μg/mL streptomycin. KGN cells were stably transduced with empty vector or RUNX3-FLAG to generate KGN/Vector and KGN/RUNX3 cells using retroviral vectors as previously described [29]. To inhibit the activity of RUNX3, we transfected COV434 cells with pcDNA3.1 or pcDNA-FLAG-RUNX3 (1-187) (kindly provided by Yoshiaki Ito, Cancer Science Institute of Singapore) using FuGene HD (Promega Corporation, Madison, WI, USA) and selected stable transformants with 1 mg/mL of G418 (Invitrogen, Waltham, MA, USA). The RUNX3 (1–187) construct expresses a truncated form of RUNX3 that contains the runt domain but lacks the transactivation domain at the carboxyl terminus. RUNX3 (1–187) functions as a dominant–negative form of RUNX3 [48,49] and was therefore referred to as dominant–negative RUNX3 (dnRUNX3) in this study.

### 4.3. Neutral Red Uptake Assay of Cell Proliferation

Cells were seeded into 96-well plates at a density of 2000 cells per well. Cell numbers on days 1, 3, 5, and 7 were determined by using the neutral red uptake assay as previously described [29]. Cell proliferation is expressed as fold-change, relative to day 1.

### 4.4. Soft Agar Assay

Cells in 2× RPMI were mixed with an equal volume of 0.7% agarose and layered above an agar base (0.5% agarose), resulting in 10,000 cells in 0.35% agar per dish. The cells were then cultured for 2–3 weeks at 37 °C and under 5% CO_2_. Colonies were stained with 1% crystal violet and counted under a microscope.

### 4.5. Cell Migration Assay

Cell migration was determined by an in vitro scratch assay as described previously [50]. Images were captured at 0 h and 24 h. The wound areas were quantified using NIH Image J software (ImageJ1, NIH, Bethesda, MD, USA) and expressed as percentages relative to 0 h.

### 4.6. Preparation of Whole Cell Lysates and Cytosolic/Nuclear Fraction and Immunoblotting

Whole cell lysates were prepared in a RIPA buffer as described previously [51]. Cytosolic and nuclear fractions were prepared using the NE-PER Nuclear and Cytoplasmic Extraction Reagents kit (Thermo Scientific, Rockford, IL, USA). Proteins in these samples were quantified using the DC protein assay (Bio-Rad, Mississauga, ON, Canada) and equal amounts of proteins were loaded into each lane for immunoblotting. Membranes were scanned and images were analyzed and quantified using an Odyssey^®^ IR scanner and Odyssey^®^ imaging software 3.0 (LI-COR Biosciences, Lincoln, NE, USA).

### 4.7. RNA Isolation and Quantitative Reverse Transcription-PCR (qRT-PCR)

RNA isolation and qRT-PCR were performed as described previously [52]. PCR primer sequences are shown in Supplementary Materials, Table S1.

### 4.8. Xenograft Model of KGN Cells

The animal experiment was conducted with the approval of the University of Alberta Health Sciences Animal Care (AUP00001435, approved on 12 May 2015) and Use Committee in accordance with guidelines from the Canadian Council for Animal Care. KGN/Vector and KGN/RUNX3 cells (2 × 10^7^ cells) were injected subcutaneously into the left and right flank, respectively, of female NSG (NOD-*scid* IL2R-gamma^null^) mice (7.5-week old, *n* = 6) twice over an interval of two weeks as described by Kim et al. [37]. Tumor formation was monitored twice a week by palpation for 170 days.

### 4.9. Expression of RUNX Proteins in GCT Tissues

Institutional approval for research with human materials was received prior to the acquisition of the GCT tissues for this study (Health Research Ethics Board of Alberta Cancer Committee, #25132 and Baylor College of Medicine IRB H-23139 and H-14435; approved on 18 February 2011, 12 May 2008, and 11 November 2003, respectively). Eleven human AGCT frozen tissues were obtained from the Alberta Cancer Research Biobank at the Cross Cancer Institute and six human AGCT, five JGCT, and two normal ovary samples were obtained from tissue repositories from the Department of Pathology at Texas Children’s Hospital and the Human Tissue Acquisition and Pathology Core at Baylor College of Medicine. Tissues were homogenized for tissue lysates collection and RNA isolation. The expression of RUNX proteins in these samples was examined by immunoblotting and qRT-PCR.

### 4.10. Statistics

Data are shown as mean ± SE of at least three independent experiments. Statistical analysis was performed using GraphPad Prism 5 (GraphPad Software, La Jolla, CA, USA). Statistical significance between the two groups was determined by a paired *t*-test and defined as *p* < 0.05.

## Supplementary Materials

Supplementary materials can be found at https://www.mdpi.com/1422-0067/20/14/3471/s1.

## References

[1] Colombo N., Parma G., Zanagnolo V., Insinga A. (2007). Management of ovarian stromal cell tumors. J. Clin. Oncol..

[2] Sekkate S., Kairouani M., Serji B., M’Rabti H., El Ghissassi I., Errihani H. (2014). Granulosa cell tumors of the ovary. Bull. Cancer.

[3] Levin G., Zigron R., Haj-Yahya R., Matan L.S., Rottenstreich A. (2018). Granulosa cell tumor of ovary: A systematic review of recent evidence. Eur. J. Obstet. Gynecol. Reprod. Biol..

[4] Jamieson S., Fuller P.J. (2012). Molecular pathogenesis of granulosa cell tumors of the ovary. Endocr. Rev..

[5] Mangili G., Ottolina J., Gadducci A., Giorda G., Breda E., Savarese A., Candiani M., Frigerio L., Scarfone G., Pignata S. (2013). Long-term follow-up is crucial after treatment for granulosa cell tumours of the ovary. Br. J. Cancer.

[6] Farkkila A., Haltia U.M., Tapper J., McConechy M.K., Huntsman D.G., Heikinheimo M. (2017). Pathogenesis and treatment of adult-type granulosa cell tumor of the ovary. Ann. Med..

[7] Shah S.P., Kobel M., Senz J., Morin R.D., Clarke B.A., Wiegand K.C., Leung G., Zayed A., Mehl E., Kalloger S.E. (2009). Mutation of foxl2 in granulosa-cell tumors of the ovary. N. Engl. J. Med..

[8] Rosario R., Cohen P.A., Shelling A.N. (2014). The role of foxl2 in the pathogenesis of adult ovarian granulosa cell tumours. Gynecol. Oncol..

[9] Mancari R., Portuesi R., Colombo N. (2014). Adult granulosa cell tumours of the ovary. Curr. Opin. Oncol..

[10] Yanagida S., Anglesio M.S., Nazeran T.M., Lum A., Inoue M., Iida Y., Takano H., Nikaido T., Okamoto A., Huntsman D.G. (2017). Clinical and genetic analysis of recurrent adult-type granulosa cell tumor of the ovary: Persistent preservation of heterozygous c.402c>g foxl2 mutation. PLoS ONE.

[11] Goyama S., Schibler J., Cunningham L., Zhang Y., Rao Y., Nishimoto N., Nakagawa M., Olsson A., Wunderlich M., Link K.A. (2013). Transcription factor runx1 promotes survival of acute myeloid leukemia cells. J. Clin. Investig..

[12] Akech J., Wixted J.J., Bedard K., van der Deen M., Hussain S., Guise T.A., van Wijnen A.J., Stein J.L., Languino L.R., Altieri D.C. (2010). Runx2 association with progression of prostate cancer in patients: Mechanisms mediating bone osteolysis and osteoblastic metastatic lesions. Oncogene.

[13] Huang B., Qu Z., Ong C.W., Tsang Y.H., Xiao G., Shapiro D., Salto-Tellez M., Ito K., Ito Y., Chen L.F. (2012). Runx3 acts as a tumor suppressor in breast cancer by targeting estrogen receptor alpha. Oncogene.

[14] Ogawa E., Inuzuka M., Maruyama M., Satake M., Naito-Fujimoto M., Ito Y., Shigesada K. (1993). Molecular cloning and characterization of pebp2 beta, the heterodimeric partner of a novel drosophila runt-related DNA binding protein pebp2 alpha. Virology.

[15] Jo M., Curry T.E. (2006). Luteinizing hormone-induced runx1 regulates the expression of genes in granulosa cells of rat periovulatory follicles. Mol. Endocrinol..

[16] Liu J., Park E.S., Jo M. (2009). Runt-related transcription factor 1 regulates luteinized hormone-induced prostaglandin-endoperoxide synthase 2 expression in rat periovulatory granulosa cells. Endocrinology.

[17] Park E.S., Lind A.K., Dahm-Kahler P., Brannstrom M., Carletti M.Z., Christenson L.K., Curry T.E., Jo M. (2010). Runx2 transcription factor regulates gene expression in luteinizing granulosa cells of rat ovaries. Mol. Endocrinol..

[18] Park E.S., Park J., Franceschi R.T., Jo M. (2012). The role for runt related transcription factor 2 (runx2) as a transcriptional repressor in luteinizing granulosa cells. Mol. Cell. Endocrinol..

[19] Ojima F., Saito Y., Tsuchiya Y., Ogoshi M., Fukamachi H., Inagaki K., Otsuka F., Takeuchi S., Takahashi S. (2019). Runx3 regulates folliculogenesis and steroidogenesis in granulosa cells of immature mice. Cell Tissue Res..

[20] Ojima F., Saito Y., Tsuchiya Y., Kayo D., Taniuchi S., Ogoshi M., Fukamachi H., Takeuchi S., Takahashi S. (2016). Runx3 transcription factor regulates ovarian functions and ovulation in female mice. J. Reprod. Dev..

[21] Wilson K., Park J., Curry T.E., Mishra B., Gossen J., Taniuchi I., Jo M. (2016). Core binding factor-beta knockdown alters ovarian gene expression and function in the mouse. Mol. Endocrinol..

[22] Lee-Thacker S., Choi Y., Taniuchi I., Takarada T., Yoneda Y., Ko C., Jo M. (2018). Core binding factor beta expression in ovarian granulosa cells is essential for female fertility. Endocrinology.

[23] Ito Y., Bae S.C., Chuang L.S. (2015). The runx family: Developmental regulators in cancer. Nat. Rev. Cancer.

[24] Lee C.W., Chuang L.S., Kimura S., Lai S.K., Ong C.W., Yan B., Salto-Tellez M., Choolani M., Ito Y. (2011). Runx3 functions as an oncogene in ovarian cancer. Gynecol. Oncol..

[25] Keita M., Bachvarova M., Morin C., Plante M., Gregoire J., Renaud M.C., Sebastianelli A., Trinh X.B., Bachvarov D. (2013). The runx1 transcription factor is expressed in serous epithelial ovarian carcinoma and contributes to cell proliferation, migration and invasion. Cell Cycle.

[26] Wang Z.Q., Keita M., Bachvarova M., Gobeil S., Morin C., Plante M., Gregoire J., Renaud M.C., Sebastianelli A., Trinh X.B. (2013). Inhibition of runx2 transcriptional activity blocks the proliferation, migration and invasion of epithelial ovarian carcinoma cells. PLoS ONE.

[27] Nevadunsky N.S., Barbieri J.S., Kwong J., Merritt M.A., Welch W.R., Berkowitz R.S., Mok S.C. (2009). Runx3 protein is overexpressed in human epithelial ovarian cancer. Gynecol. Oncol..

[28] Greer A.H., Yong T., Fennell K., Moustafa Y.W., Fowler M., Galiano F., Ng S.W., Berkowitz R.S., Cardelli J., Meyers S. (2013). Knockdown of core binding factorbeta alters sphingolipid metabolism. J. Cell. Physiol..

[29] Barghout S.H., Zepeda N., Vincent K., Azad A.K., Xu Z., Yang C., Steed H., Postovit L.M., Fu Y. (2015). Runx3 contributes to carboplatin resistance in epithelial ovarian cancer cells. Gynecol. Oncol..

[30] Dhillon V.S., Shahid M., Husain S.A. (2004). Cpg methylation of the fhit, fancf, cyclin-d2, brca2 and runx3 genes in granulosa cell tumors (gcts) of ovarian origin. Mol. Cancer.

[31] Salto-Tellez M., Peh B.K., Ito K., Tan S.H., Chong P.Y., Han H.C., Tada K., Ong W.Y., Soong R., Voon D.C. (2006). Runx3 protein is overexpressed in human basal cell carcinomas. Oncogene.

[32] Anttonen M., Pihlajoki M., Andersson N., Georges A., L’Hote D., Vattulainen S., Farkkila A., Unkila-Kallio L., Veitia R.A., Heikinheimo M. (2014). Foxl2, gata4, and smad3 co-operatively modulate gene expression, cell viability and apoptosis in ovarian granulosa cell tumor cells. PLoS ONE.

[33] Cheng J.C., Chang H.M., Qiu X., Fang L., Leung P.C. (2014). Foxl2-induced follistatin attenuates activin a-stimulated cell proliferation in human granulosa cell tumors. Biochem. Biophys. Res. Commun..

[34] Wang C., Lv X., Jiang C., Cordes C.M., Fu L., Lele S.M., Davis J.S. (2012). Transforming growth factor alpha (tgfalpha) regulates granulosa cell tumor (gct) cell proliferation and migration through activation of multiple pathways. PLoS ONE.

[35] Robker R.L., Richards J.S. (1998). Hormone-induced proliferation and differentiation of granulosa cells: A coordinated balance of the cell cycle regulators cyclin d2 and p27kip1. Mol. Endocrinol..

[36] Imai M., Muraki M., Takamatsu K., Saito H., Seiki M., Takahashi Y. (2008). Spontaneous transformation of human granulosa cell tumours into an aggressive phenotype: A metastasis model cell line. BMC Cancer.

[37] Kim J.H., Kim Y.H., Kim H.M., Park H.O., Ha N.C., Kim T.H., Park M., Lee K., Bae J. (2014). Foxl2 posttranslational modifications mediated by gsk3beta determine the growth of granulosa cell tumours. Nat. Commun..

[38] Bilandzic M., Wang Y., Ahmed N., Luwor R.B., Zhu H.J., Findlay J.K., Stenvers K.L. (2014). Betaglycan blocks metastatic behaviors in human granulosa cell tumors by suppressing nfkappab-mediated induction of mmp2. Cancer Lett..

[39] Goh Y.M., Cinghu S., Hong E.T., Lee Y.S., Kim J.H., Jang J.W., Li Y.H., Chi X.Z., Lee K.S., Wee H. (2010). Src kinase phosphorylates RUNX3 at tyrosine residues and localizes the protein in the cytoplasm. J. Biol. Chem..

[40] Chi X.Z., Kim J., Lee Y.H., Lee J.W., Lee K.S., Wee H., Kim W.J., Park W.Y., Oh B.C., Stein G.S. (2009). Runt-related transcription factor RUNX3 is a target of MDM2-mediated ubiquitination. Cancer Res..

[41] Jin Y.H., Jeon E.J., Li Q.L., Lee Y.H., Choi J.K., Kim W.J., Lee K.Y., Bae S.C. (2004). Transforming growth factor-beta stimulates p300-dependent RUNX3 acetylation, which inhibits ubiquitination-mediated degradation. J. Biol. Chem..

[42] Hnit S.S., Xie C., Yao M., Holst J., Bensoussan A., De Souza P., Li Z., Dong Q. (2015). P27(kip1) signaling: Transcriptional and post-translational regulation. Int. J. Biochem. Cell Biol..

[43] Haltia U.M., Andersson N., Yadav B., Farkkila A., Kulesskiy E., Kankainen M., Tang J., Butzow R., Riska A., Leminen A. (2017). Systematic drug sensitivity testing reveals synergistic growth inhibition by dasatinib or mtor inhibitors with paclitaxel in ovarian granulosa cell tumor cells. Gynecol. Oncol..

[44] Chan-Penebre E., Armstrong K., Drew A., Grassian A.R., Feldman I., Knutson S.K., Kuplast-Barr K., Roche M., Campbell J., Ho P. (2017). Selective killing of smarca2- and smarca4-deficient small cell carcinoma of the ovary, hypercalcemic type cells by inhibition of ezh2: In vitro and in vivo preclinical models. Mol. Cancer Therapeutics.

[45] Carlton A.L., Illendula A., Gao Y., Llaneza D.C., Boulton A., Shah A., Rajewski R.A., Landen C.N., Wotton D., Bushweller J.H. (2018). Small molecule inhibition of the cbfbeta/runx interaction decreases ovarian cancer growth and migration through alterations in genes related to epithelial-to-mesenchymal transition. Gynecol. Oncol..

[46] Nishi Y., Yanase T., Mu Y., Oba K., Ichino I., Saito M., Nomura M., Mukasa C., Okabe T., Goto K. (2001). Establishment and characterization of a steroidogenic human granulosa-like tumor cell line, kgn, that expresses functional follicle-stimulating hormone receptor. Endocrinology.

[47] Zhang H., Vollmer M., De Geyter M., Litzistorf Y., Ladewig A., Durrenberger M., Guggenheim R., Miny P., Holzgreve W., De Geyter C. (2000). Characterization of an immortalized human granulosa cell line (cov434). Mol. Hum. Reproduct..

[48] Ito K., Liu Q., Salto-Tellez M., Yano T., Tada K., Ida H., Huang C., Shah N., Inoue M., Rajnakova A. (2005). Runx3, a novel tumor suppressor, is frequently inactivated in gastric cancer by protein mislocalization. Cancer Res..

[49] Yano T., Ito K., Fukamachi H., Chi X.Z., Wee H.J., Inoue K., Ida H., Bouillet P., Strasser A., Bae S.C. (2006). The runx3 tumor suppressor upregulates bim in gastric epithelial cells undergoing transforming growth factor beta-induced apoptosis. Mol. Cell. Biol..

[50] Xu Z., Jiang Y., Steed H., Davidge S., Fu Y. (2010). Tgfbeta and egf synergistically induce a more invasive phenotype of epithelial ovarian cancer cells. Biochem. Biophys. Res. Commun..

[51] Fu Y., Sies H., Lei X.G. (2001). Opposite roles of selenium-dependent glutathione peroxidase-1 in superoxide generator diquat- and peroxynitrite-induced apoptosis and signaling. J. Biol. Chem..

[52] Azad A.K., Chakrabarti S., Xu Z., Davidge S.T., Fu Y. (2014). Coiled-coil domain containing 3 (ccdc3) represses tumor necrosis factor-alpha/nuclear factor kappab-induced endothelial inflammation. Cell. Signall..

