# Supplementary material for: RUNX3 Promotes the Tumorigenic Phenotype in KGN, a Human Granulosa Cell Tumor-Derived Cell Line
Source: Int J Mol Sci. 2019 Jul 15;20(14):3471. doi: 10.3390/ijms20143471 (PMC6678151; doi:10.3390/ijms20143471)
Supplement: Supplementary file 1 [file ijms-20-03471-s001.pdf]

Supplementary Figure 1

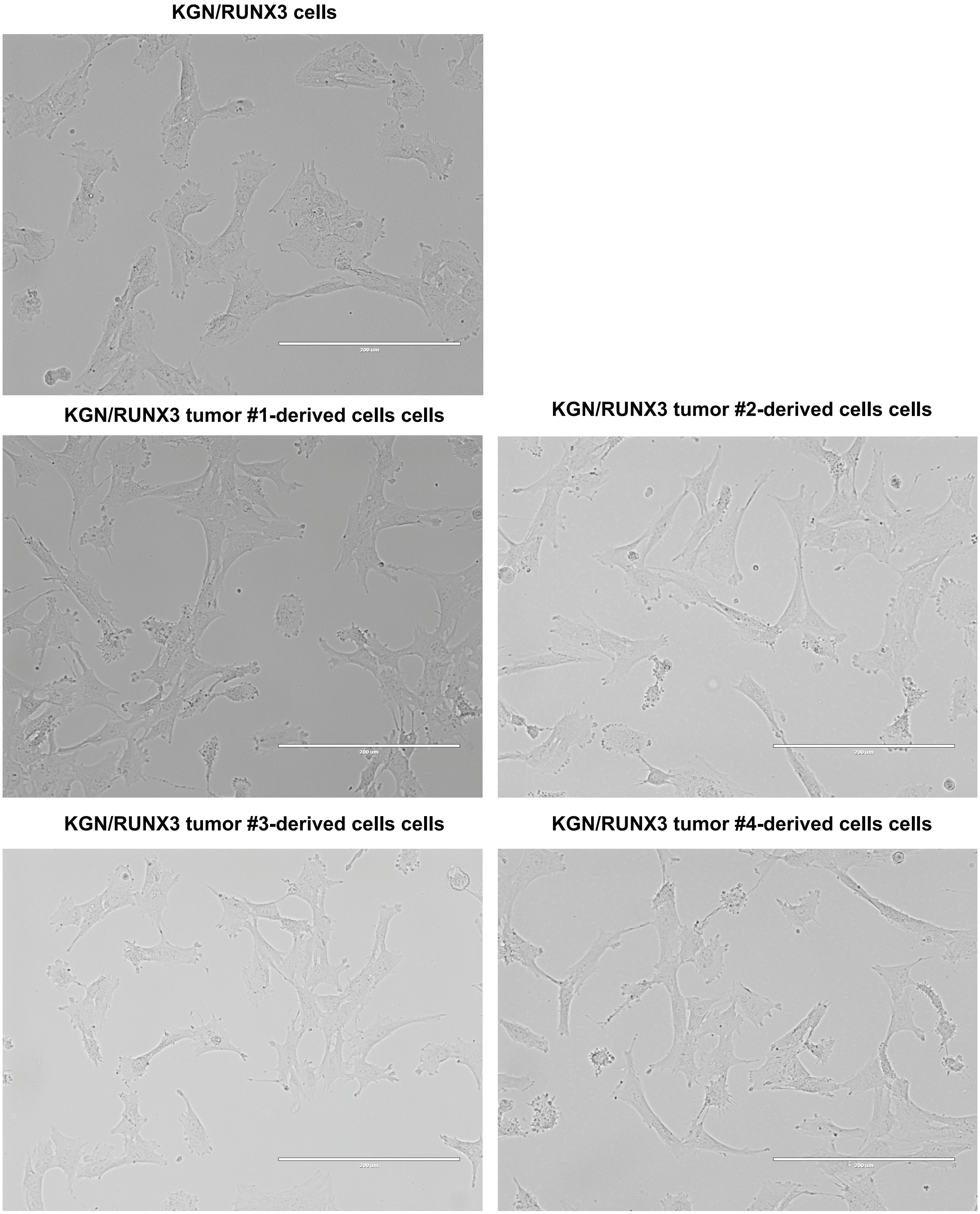

Supplementary Figure 1. Images of cells derived from the xenografted KGN/RUNX3 tumors. Representative images of KGN/RUNX3 cells and cells derived from the xenografted KGN/RUNX3 tumors are shown. Scale bar = 200 μm. Cells derived from the KGN/RUNX3 tumors show the same morphology as the KGN/RUNX3 cells used for injection.

Supplementary Table 1. Primer sequences

Genes	Forward primer	Reverse primer
CNDN2	TTCCCTCTGGCCATGAATTAC	GGGCTGGTCTCTTTGAGTTT
CDKN1B	CTAACTCTGAGGACACGCATTT	TGCAGGTCGCTTCCTTATTC
RUNX2	ACGAATGCACTATCCAGCCACCTT	ATATGGAGTGCTGCTGGTCTGGAA
RUNX3	TGGCAGGCAATGACGAGAACTACT	TGAACACAGTGATGGTCAGGGTGA
GAPDH	GGACCTGACCTGCCGTCTAGAA	GGTGTCGCTGTTGAAGTCAGAG
